# Associations of appendicular lean mass and abdominal adiposity with insulin resistance in older adults: A cross-sectional study

**DOI:** 10.1371/journal.pone.0303874

**Published:** 2024-05-16

**Authors:** Justin J. Cheng, Li-Jung Liang, Cathy C. Lee

**Affiliations:** 1 Geriatric Research Education and Clinical Center, Veterans Affairs Greater Los Angeles Healthcare System, Los Angeles, California, United States of America; 2 Division of General Internal Medicine and Health Services Research, David Geffen School of Medicine at UCLA, Los Angeles, California, United States of America; 3 David Geffen School of Medicine at UCLA, Los Angeles, California, United States of America; University of Campania Luigi Vanvitelli: Universita degli Studi della Campania Luigi Vanvitelli, ITALY

## Abstract

Loss of lean muscle mass and accumulation of adipose tissue are changes associated with aging. Previous studies have documented various components of body composition as predictors for insulin resistance. The objective of this study was to investigate whether components of body composition—appendicular lean mass (ALM) and/or abdominal fat mass (AFM)—correlate with insulin resistance in older men and women. This was a cross-sectional study of 92 older men and women. Weight was classified according to body mass index (BMI)–normal (BMI <25), overweight (BMI 25–30), and obese (BMI >30). All body composition data was determined by dual energy x-ray absorptiometry (DEXA), and insulin resistance was assessed by the homeostatic model assessment of insulin resistance (HOMA-IR). Multivariable regression models with two-way interaction terms were employed to assess whether the associations between components of body composition and log HOMA varied by BMI categories. Adjusted regression showed that log HOMA was significantly associated with AFM (estimate ± standard error: 0.055 ± 0.026) and ALM (0.057 ± 0.029) for the overweight participants (p-values <0.05). Additionally, the adjusted associations between log HOMA and ALM were significantly greater for participants who were either obese or overweight compared to those with a normal BMI (p<0.002). Less consistent relationships were observed between insulin resistance and abdominal fat mass across BMI categories, whereas more consistent associations were observed between insulin resistance and appendicular lean mass in individuals with greater BMI. Further research is needed to clarify if lipid deposition within muscle tissue promotes muscle dysfunction and thereby increases risk for insulin resistance.

## Introduction

While obesity has long been implicated in the development of insulin resistance, it is now known that decreased muscle mass or sarcopenia also plays a role [1–5]. Typical aging-associated changes of body composition include loss of lean muscle mass and accumulation of adipose tissue [6]. Sarcopenia, coupled with increased adiposity has many consequences in an older population such as compromised strength, functional limitation, and decreased mobility [7]. In addition, skeletal muscle is one of the primary sites of glucose uptake in response to the release of insulin by the pancreas [8, 9]. Therefore, a decrease in skeletal muscle mass could contribute to decreased insulin sensitivity.

While still conventionally used in clinical settings and population studies, body mass index (BMI) as a sole estimate may not accurately capture body composition. Overweight individuals may have increases in both fat and muscle mass. Increases in just muscle mass can all lead to a higher BMI. Dual-energy x-ray absorptiometry (DEXA) allows for easy and cost-effective quantitative measurement of body composition. In a study of younger adults assessing body composition models, individuals with high adiposity and low muscle mass exhibited lower insulin sensitivity [10]. Thus, developing a definitive model in older adults correlating components of body composition and insulin resistance is crucial for exploring therapeutic interventions that can potentially lower risk for development of diabetes. While adiposity has shown independent effects on insulin resistance, the contribution of sarcopenia suggests that a model encompassing both sarcopenia and components of adiposity is important for predicting development of type 2 diabetes [9–13].

The present study examined the correlation of various components of body composition with insulin resistance in older men and women. We explore these associations to suggest a model that accounts for the influence of both lean and fat mass on insulin resistance. Our hypothesis was that decreased appendicular lean mass (ALM) or increased abdominal fat mass (AFM) is best correlated with insulin resistance in older men and women.

## Materials and methods

### Study population

Secondary analysis of de-identified data from 65 healthy postmenopausal women and 27 healthy older men were included. Subjects were screened prior to study enrollment based on medical history; physical examination; and laboratory tests, including a complete blood count, thyroid function tests and routine chemistries, and an oral glucose tolerance test (OGTT). Exclusion criteria included diabetes mellitus (based on 75-gram OGTT) or evidence of other significant underlying medical or psychiatric illness based on history, physical examination, and laboratory testing.

### Measurement of insulin sensitivity

Blood samples for glucose and insulin were collected in glass tubes containing sodium heparin, stored on ice, and separated immediately after collection. Plasma was stored at -70°C until assay. Plasma glucose was measured using the glucose oxidase method and plasma insulin by radioimmunoassay (RIA) and immulite analyzer. Insulin sensitivity was determined using the homeostatic assessment method (HOMA) calculated by the equation:

$$Insulin_{fasting}(\text{μU/mL})\times \frac{Glucose_{fasting}(\text{mmol/L})}{22.5}$$

### Measurement of body composition

**Fig 1:**
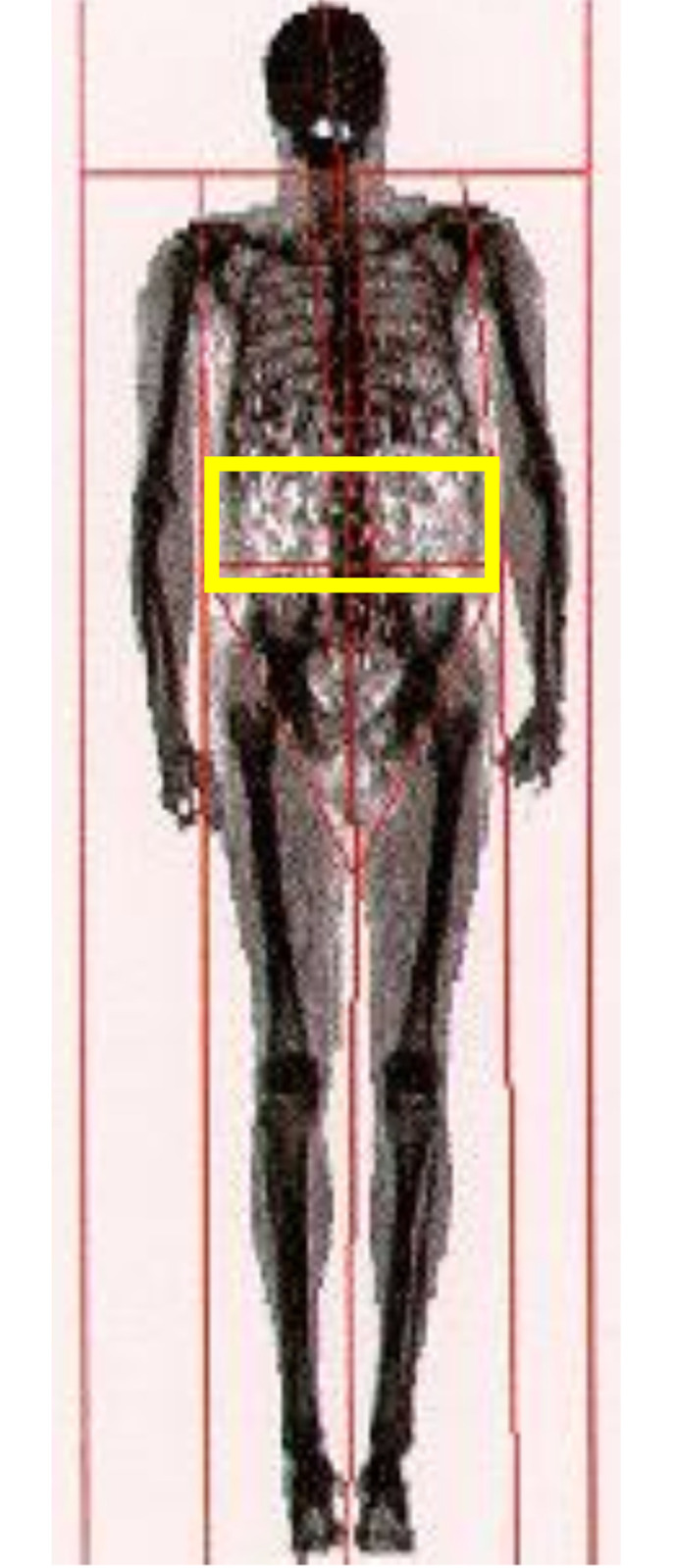
Measurement of body composition via dual x-ray absorptiometry (DEXA). A rectangle was drawn on the digital image bounded superiorly by a horizontal line identifying the T12/L1 intervertebral space, inferiorly by a horizontal line denoting the L4/L5 intervertebral space, and bilaterally by connecting the two horizontal lines in a region free of tissue. Abdominal adiposity, DXA L1-L4, was defined as the fat mass within this region.

Lean body mass and total body composition were determined by dual energy x-ray absorptiometry (DEXA) (model DPX-L; Lunar Radiation, Madison, WI) as previously described [14, 15]. The DEXA measurement of abdominal adiposity (DEXA L1-L4) or central fat mass was determined manually. A rectangle was drawn on the digital image bounded superiorly by a horizontal line identifying the T12/L1 intervertebral space, inferiorly by a horizontal line denoting the L4/L5 intervertebral space, and bilaterally by connecting the two horizontal lines in a region free of tissue. Abdominal adiposity, DXA L1-L4, was defined as the fat mass within this region (Fig 1) [14, 15].

### Statistical analysis

Sample characteristics and measures of interest were described by means, standard deviations (SD), and frequencies. Multivariable linear regression model with two-way interaction terms was performed to assess whether the associations across different BMI categories were varied through model contrasts. Models included age, gender, BMI category (normal, overweight, and obese), and two-way interactions with BMI category (i.e., AFM-by-BMI and ALM-by-BMI). Data analysis was performed using SAS version 9.4 (SAS Institute, Inc., Cary, NC). Statistical significance was defined as p <0.05.

### Ethics

The study was approved by the Greater Los Angeles VA Institutional Review Board (IRB) and was compliant with the Health Insurance Portability and Accountability Act (IRB #1615836–4). Each subject gave written informed consent prior to enrollment in the initial study. Data on archived samples was accessed on December 1, 2023, to conduct the secondary analysis. The authors had no access to information that could identify individual participants after data collection.

## Results

Baseline characteristics of the participants are illustrated in Table 1. Over 70% of study participants were female, and the average age for female participants was 5.3 years older than that for male participants (71.7 vs. 66.4, p = 0.002). According to BMI, 40% of participants were overweight, 23% obese, and 36% normal. There was only one participant categorized as underweight.

**Table 1:** Baseline characteristics of participants (N = 92).

Characteristic	Mean (SD) or N (%)
Age (years)	70.1 (7.3) (range 60–86)
Female	65 (70.7%)
Body mass index (kg/m2)	26.8 (4.6)
Body mass index (BMI) category	
Underweight (BMI <19)	1 (1%)
Normal (BMI 19–25)	33 (37%)
Overweight (BMI 25–30)	37 (40%)
Obese (BMI >30)	21 (23%)
Abdominal fat mass (kg)	12.5 (5.3)
Total fat mass (kg)	25.3 (9.4)
Percent body fat (%)	34.8 (11.6)
Appendicular lean mass (kg)	18.9 (6.0)
Glucose (mg/dL)	95.3 (13.1)
Insulin (μU/mL)	11.3 (6.8)
HOMA-IR (log)	2.8 (1.8)

HOMA-IR = homeostatic model assessment of insulin resistance.

The average value of Homeostatic Model Assessment of Insulin Resistance (HOMA-IR) value was 2.75 (SD 1.76). Female participants had a significantly greater mean value of HOMA-IR than male participants (female, 3.07±1.55; male, 2.01±2.04, p = 0.008). The mean HOMA-IR for individuals classified as obese was the highest among the three BMI categories (1.97±1.13, 2.98±2.80, and 3.55±1.10 for normal, overweight, and obese groups, respectively).

Results from multivariable regression showed a significant association between log HOMA and AFM for the overweight participants (estimate ± standard error (SE): 0.055 ± 0.026, p = 0.037); however, this association was not significantly different across the three BMI categories (p = 0.077) (Table 2).

**Table 2:** Multivariable regression model on homeostatic model assessment of insulin resistance (HOMA-IR, log scale).

Covariate	Estimate	SE	P Value
Age	0.005	0.009	0.557
Female	**1.173**	**0.300**	**<0.001**
Body Mass Index (Reference = Obese)			
Normal	0.605	0.694	0.386
Overweight	-0.668	0.781	0.395
Abdominal Fat Mass (kg)			
Normal	0.047	0.028	0.097
Overweight	**0.055**	**0.026**	**0.037**
Obese	0.005	0.022	0.826
Appendicular Lean Mass (kg)			
Normal	-0.036	0.029	0.218
Overweight	**0.057**	**0.029**	**0.049**
Obese	**0.060**	**0.027**	**0.030**

SE = Standard error

**Fig 2:**
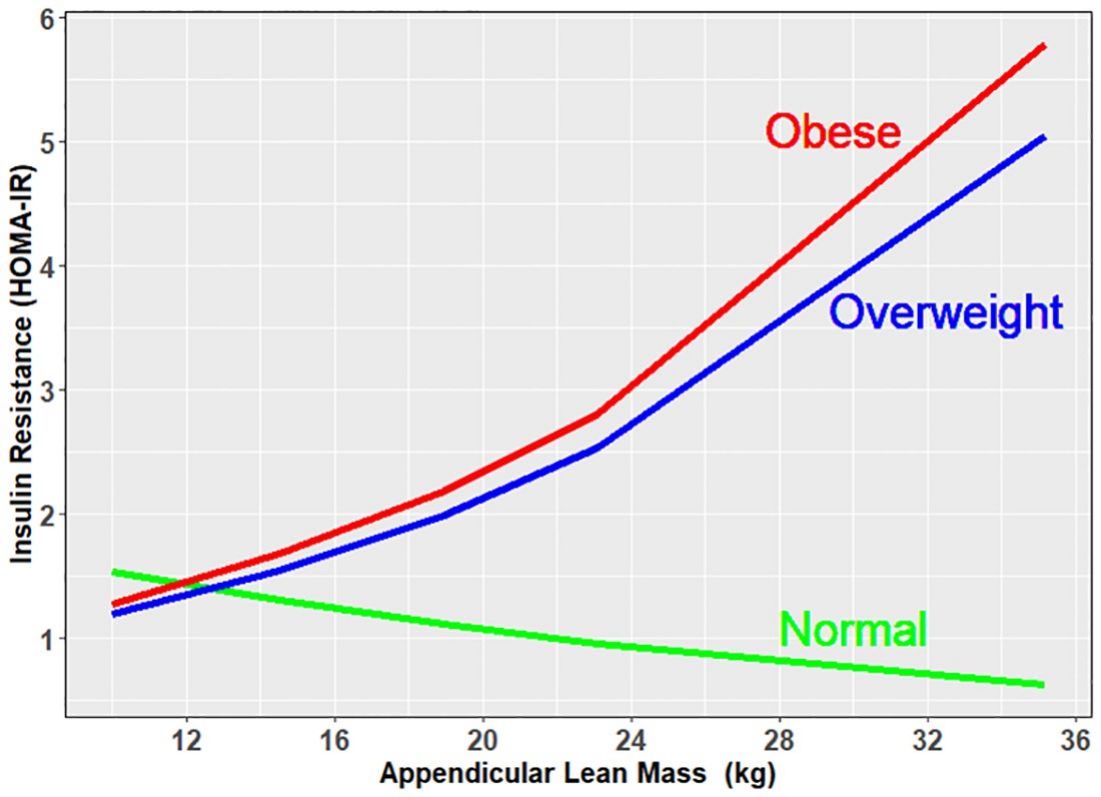
Adjusted association between insulin resistance (HOMA-IR) and appendicular lean mass by BMI category.

The adjusted associations between log HOMA and ALM were significantly greater for participants who were either obese or overweight compared to those with a normal BMI (difference between obese and normal ± SE: 0.093 ± 0.028; overweight and normal, 0.095 ± 0.028; p< 0.002). The relationship of these associations is shown in Fig 2.

## Discussion

The present study demonstrated that in older adults, abdominal fat mass exhibited a positive, significant relationship with insulin resistance in overweight individuals. Contrary to our initial hypothesis, this correlation between AFM and insulin resistance was not found in normal BMI or obese participants. Also contrary to our initial thoughts, appendicular lean mass showed a positive correlation with insulin resistance in overweight and obese individuals.

Previous studies have shown that older individuals with increased AFM are more likely to be insulin resistant [15, 16]. In the present study, the relationship between body composition and insulin resistance varied among normal weight, overweight, and obese older adults. ALM was significantly associated with insulin resistance in overweight and obese individuals. This is similar to a longitudinal, prospective cohort study where an association between appendicular lean mass and metabolic syndrome was found [4]. A cross-sectional study also found that decreased lower limb muscle mass and appendicular muscle mass was associated with increased insulin resistance [17]. The presence of sarcopenia can coexist with low bone density, known as osteosarcopenia, which is known to be associated with insulin resistance and type 2 diabetes mellitus [18]. In normal weight individuals (BMI < 25), neither ALM nor AFM were significantly correlated with insulin resistance. These findings suggest that in normal weight individuals, lean and fat mass are not associated with insulin resistance in the same manner as in overweight individuals. Because skeletal muscle contains one of the primary sites for glucose uptake, it might be expected that higher muscle mass would correlate with increased insulin sensitivity [8, 19]. However, in the current study, there was a positive correlation between skeletal muscle and insulin resistance in overweight and obese individuals. Additionally, AFM was not associated with insulin resistance in obese individuals, which contradicts prior studies that primarily focused on the role of adipose tissue in insulin resistance [15, 16]. This seems to suggest that in individuals with higher BMI, appendicular lean mass may play a greater role in risk for insulin resistance.

The positive association between skeletal muscle mass and insulin resistance in overweight and obese individuals is consistent with prior studies, which have shown that sarcopenia contributes more to insulin resistance in older adults than sarcopenic obesity or obesity alone [3, 4, 12, 17]. It is known that higher lean mass is often accompanied by higher fat mass in older adults [20, 21]. There may be biochemical mechanisms that explain this phenomenon, including increased lipid deposition within myotubules contributing to the overexpression of muscle lipoprotein lipase [22, 23]. Muscle lipoprotein lipase has been associated with insulin resistance, as has accumulation of lipids inside muscle cells [24, 25]. Adipose tissue deposition between muscle cells, described as intermuscular adipose tissue (IMAT), also can play a role in metabolic syndrome and insulin resistance [26]. IMAT content increases with age, and this can contribute to decreased insulin sensitivity [27]. Altogether, these biologic phenomena may explain the relationship between increased muscle mass and insulin resistance.

Another explanation for the association between lean mass and insulin resistance might be the heterogeneity of skeletal muscle, which includes both Type I and Type II muscle fibers. Type I fibers are typically more responsive to insulin than Type II fibers, and overweight individuals tend to exhibit increased type II fibers and fewer type I fibers [28]. Although it is generally felt that higher skeletal muscle mass correlates with increased insulin sensitivity, the findings of our present study might suggest that the observed decrease in insulin sensitivity could be secondary to an increased proportion of type II fibers.

There are several limitations to the present study. First, the study subjects were healthy, community dwelling older adults and therefore, the conclusions from the study cannot be extrapolated to older adults who are frail, hospitalized or severely ill. Second, the use of BMI to characterize body composition may limit comparisons to other studies that utilized body fat percentage or other surrogate markers of composition. Third, our study did not utilize advanced imaging techniques (CT or MRI). Although CT and MRI can clearly distinguish adipose from muscle tissue, it is still limited in differentiating intramyocellular from intermuscular lipid deposition [26]. Finally, the cross-sectional nature of the study limits the ability to draw cause-effect inferences.

## Conclusion

**Fig 3:**
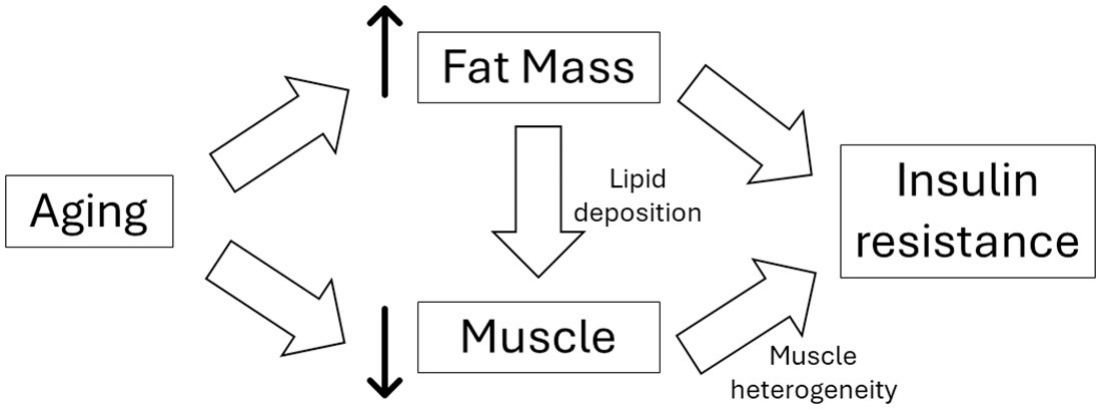
Schematic of potential pathophysiology between body composition and insulin resistance. Aging is associated with increased fat mass and decreased muscle mass. Fat mass may be related to muscle due to lipid deposition whereas muscle may be related to insulin resistance through muscle heterogeneity.

In our study of independent, community-dwelling older adults, appendicular lean mass exhibited a positive correlation with insulin resistance in overweight and obese individuals. Abdominal fat mass also exhibited an association with insulin resistance but only in overweight individuals. Based on other studies, these findings may be explained by lipid deposition within or between muscle cells or the heterogeneity of muscle fibers associated with aging (Fig 3).

Gender could also play a role given the different physiological distribution of fat and muscle between males and females. Altogether, this highlights the need for further research assessing the quality of muscle mass in older adults and the potential implication of muscle dysfunction leading to insulin resistance. Perhaps, in older individuals, developing quality muscle mass is more important than loss of fat to decrease risk for insulin resistance and diabetes mellitus.
